# Motion Analysis for Jumping Discus Throwing Correction

**DOI:** 10.3390/ijerph182413414

**Published:** 2021-12-20

**Authors:** Chao-Fu Chen, Hui-Ju Wu, Zheng-Sheng Yang, Hui Chen, Hsien-Te Peng

**Affiliations:** 1 Physical Education College, Huaibei Normal University, Huaibei 235000, China; chenchaofu@chnu.edu.cn (C.-F.C.); wuhuiru@chnu.edu.cn (H.-J.W.); 2 Graduate Institute of Sport Coaching Science, Chinese Culture University, Taipei 11114, Taiwan; 3 Department of Physical Education, Chinese Culture University, Taipei 11114, Taiwan; socceryjs@yahoo.com.tw (Z.-S.Y.); chenhui5417@yahoo.com.tw (H.C.)

**Keywords:** biomechanics, sport performance, track and field, kinematics

## Abstract

The purpose of this study was to explore the kinematical characteristics of jumping discus throwing. Eight male right-handed discus throwers who used to practice the jumping throwing technique were recruited as participants. Two high-speed digital cameras with 120 Hz sampling rate were synchronized to capture the movement. The captured images were processed using a motion analysis suite, and the markers attached to joints on images were digitized manually. Based on the results, throwers should keep smaller the shoulder–hip twisting and the right anterior superior iliac spine (abbreviated: ASIS) in front of the right acromion (for right-handed throwers) from the instant the right foot lands to the instant the left foot lands, before the instant of the right foot lands; keep the discus at a depressed position; and reduce the time before discus release, particularly the time of the non-support phase and the second single-support phase. Additionally, release velocity must be improved because throwing distance is directly proportional to squared release velocity. In conclusion, the current study demonstrated comprehensive kinematical analyses, which can be used to instruct the jumping discus throwing technique with duration and angle characteristics of throwing movement for athletes by coaches with videos.

## 1. Introduction

Discus throw is a track and field sport that involves throwing a heavy discus in a spinning movement. The athlete moves in a manner similar to hammer throw or rotational shot put but spins the body and releases the object differently [1,2]. Researchers have noted that although the spinning movement performed before the release of the discus is essentially the same among discus throwers, the action performed following the instant of release of the discus can be divided into two categories: airborne-release and grounded-release. Airborne-release refers to the athlete releasing the discus while in the air (i.e., with both feet above the ground), and grounded-release refers to the athlete releasing the discus with one or both feet on the ground [3]. In some studies, these terms are replaced by “jumping” (or “rotation”) discus throwing and “double-support” (or “standing”) discus throwing, respectively [3,4].

Dapena [5] investigated the force–energy conversion pattern of jumping discus throwing by analysing the relationship between forces, linear momentum, and rotational energy, discovering that in the initial double-support phase, a large pull–push horizontal force is generated, and at the instant of release, the linear momentum and kinetic energy generated by the thrower and the discus (regarded as two separate systems) contributed 7% and 12%, respectively, to the horizontal and vertical velocity of the discus, whereas the thrower’s trunk contributed 93% and 88% of rotational kinetic energy on the vertical and forward direction, respectively. To successfully initiate a kinetic chain to throw the discus [6,7], the thrower’s trunk must reach a high rotational angular velocity in the vertical direction when spinning, which enables the energy generated by the lower limbs during swinging and spinning to be transmitted to the trunk. At the instant of release, the extensive twisting of the shoulders and the hip enables the energy to be transmitted to the discus through the throwing arm, allowing the discus to take flight [3,5]. A study reported that athletes using jumping discus throwing had relatively greater momentum of a thrower-plus-discus system along the vertical direction, resulting in shorter discus flight time [3].

The twisting of the trunk is crucial to flight time. Other than linear momentum and rotational energy generated by the system, other key indicators include the spinning movement of the trunk and the throwing arm [8,9]. To increase throwing distance, at the instant of release, the trunk must be tilted backward (−6° on average), the discus vertical velocity must be high (13.10 m/s on average), and the shoulder–hip separation angle (47° on average) and arm–shoulder separation angle (−7° on average) must remain as stable as possible [9]. Results of movement variability (standard deviation) analysis have revealed that a less discrete distribution of standard deviation values (i.e., less variation in movement) results in longer throwing distances [10].

In previous studies, the jumping discus throwing and double-support discus throwing were pooled together in discus analyses, which may confound the results [8,9,10]. Dapena (1993) and Dapena and Anderst (1997) [3,5] have compared differences of the throwing techniques in a case study, but only kinetics or how to utilize it to perform the techniques were reported. Other studies either have not provided relevant information on jumping discus throwing or have not compared the difference between the throwing techniques [8,9,10]. Moreover, in regular training, it was difficult and time-consuming to observe or analyse previously reported kinetic variables such as angular velocity, force, momentum, and rotational energy of the discus throwing [3,5,6,7]. Therefore, the current study aimed to explore the kinematical characteristics of jumping discus throwing, which can be easily and conveniently performed with digital cameras to provide coaches and athletes with a reference for technique improvement.

## 2. Materials and Methods

### 2.1. Participants

Eight male discus throwers competing in their respective highest national division league were recruited (height 178.13 ± 5.57 cm; weight 109.75 ± 12.99 kg; age 22.75 ± 2.38 years; and throw distance 42.08 ± 4.89 m). All of the participants used to practice jumping discus throwing with the right arm as the throwing arm.

Before the experiment commenced, the participants were informed of the purpose of this study and were required to sign a letter of consent. The study was approved by the ethics committee of a hospital to meet the ethical standards of the institution.

### 2.2. Equipment and Data Collection 

Two high-speed digital cameras (sampling rate = 120 Hz, shutter speed = 1/1000 s, Sony, HDR-AX2000, TOKYO, Japan) were set up at locations 12 m from the centre of the throwing circle—one on the rear side and one on the lateral side. The cameras (specifically, the optical axes of their lenses) were pointed at the centre of the coordinate system setup, covering an area 2 m from the coordinate system setup and the throwing circle. One LED served as a beacon for synchronisation of the cameras. A three-dimensional coordinate frame 2.5 m × 2.0 m × 2.5 m (Length × Width × Height) (Peak Motus) in size and including 25 markers was set up with its origin at the centre of the throwing circle to construct a globe coordinate system before data collection. The participants had 19 markers attached to the head, C7, right and left shoulder, elbow, wrist, mid-finger, ASIS, knee, ankle, mid-toe joints, and discus and commenced throwing after 10 min dynamic warming up and 10 min throwing-specific warming up including two discus throws. The discuses used were 2 kg standard discuses conforming to World Athletics specifications. Per the rules of official games, six maximum-effort throws with a 10 min rest between trials were performed, and that with the greatest distance was included for analysis.

### 2.3. Data Processing

The captured images were processed using Kwon3D motion analysis suite (Visol, Inc., Gwangmyeong-si, Kyonggi-do, Korea), and the markers attached to joints on images were digitized manually. The X, Y, and Z axes of the global coordinate system represented the horizontal left–right, forward–backward, and vertical upward–downward directions of the space, respectively. The anthropometric parameters of young males were referenced [11].

**Figure 1:**
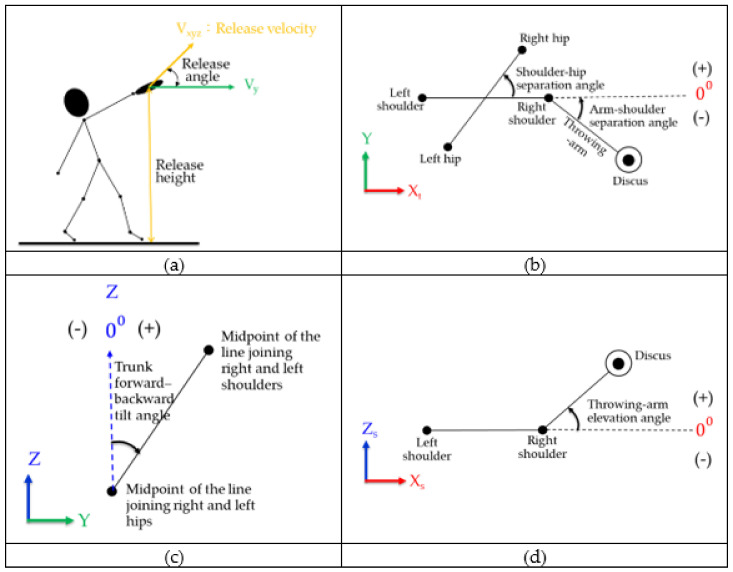
Definition of the angle parameters. (**a**) release angle, release height, and release velocity. (**b**) arm–shoulder separation angle and shoulder–hip separation angle. (**c**) trunk forward–backward tilt angle. (**d**) throwing arm elevation angle.

Additionally calculated were release velocity, which was the resultant velocity ($\vec{Rv}$) of the discus’s velocity along the X, Y, and Z axes (${\vec{V}}_{x}$, ${\vec{V}}_{y}$ and ${\vec{V}}_{z}$, respectively); release angle, which was the angle formed by ${\vec{V}}_{y}$ and $\vec{Rv}$; and release height, which was the height from the sagittal plane of the discus to the ground at the instant of release (Figure 1a). A local coordinate system was established for the trunk, with the Xt axis being the line formed by the bilateral anterior superior iliac spines (ASISs), the Yt axis being the direction of ASISs facing forward, and the Zt axis being the line formed by the midpoint of the bilateral ASISs and the midpoint of the bilateral acromia; then, the shoulder–hip separation angle (Figure 1b), arm–shoulder separation angle (Figure 1b), trunk forward–backward tilt angle (Figure 1c), and throwing arm elevation angle (Figure 1d) were calculated [9].

In the equation that follows, *V_h_*, *V_s_*, and *V_d_* represent the local vector of the vector from the left ASIS to the right ASIS, the local vector from the left acromion to the right acromion, and the local vector from the centre of the discus to the right acromion, respectively; they revolve around the XtYt plane of the trunk coordinate system. Therefore, the shoulder–hip separation angle (α) can be defined as the vector product of *Vs* and *V_h_*, with a positive value indicating that the right ASIS is in front of the right acromion and a negative value indicating that the right acromion is in front of the right ASIS (Figure 1b). The arm–shoulder separation angle (β) can be defined as the vector product of *V_d_* and *V_s_*, with a positive value indicating that the right acromion is in front of the right arm and a negative value indicating that the right arm is in front of the right acromion (Figure 1c). The throwing arm elevation angle (γ) was defined as the projection angle of the right shoulder–discus vector to the Xs axis of the shoulder’s local coordinate system on the XZ plane, with a positive value indicating that the discus was in a position higher than the right shoulder (i.e., the right shoulder was raised) and a negative value indicating that the discus was in a position lower than the right shoulder (i.e., the right shoulder was depressed) (Figure 1d) [9]. 

The trunk forward–backward tilt angle was calculated from the Cardan angles of the trunk revolving around the global coordinate system. The rotation sequence was X (extension/flexion)–Y (abduction/adduction)–Z (external rotation/internal rotation). In this study, extension–flexion was adopted as the trunk forward–backward tilt angle; when the trunk revolved around the YZ plane, the trunk tilting forward and away from the Z axis was defined as a positive value, indicating trunk forward tilt angle, and the trunk tilting backward and away from the Z axis was defined as a negative value, indicating trunk backward tilt angle [9,10]. The data on the three-dimensional space were filtered by applying the Butterworth fourth-order low-pass filter with the cut-off frequency at 6 Hz before all kinematic parameters were calculated.

**Figure 2:**
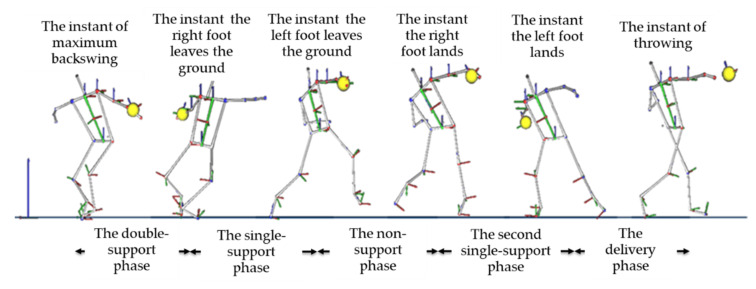
Definition of the phases.

In this study, discus throwing was divided into five phases: (1) double-support phase: from the instant of maximum backswing, which is defined as the instant of maximum shoulder–hip separation angle, to the instant the right foot leaves the ground; (2) initial single-support phase: from the instant the right foot leaves the ground to the instant the left foot leaves the ground; (3) non-support phase: from the instant the left foot leaves the ground to the instant the right foot lands; (4) second single-support phase: from the instant the right foot lands to the instant the left foot lands; and (5) delivery phase: from the instant the left foot lands to the instant the discus releases from the participant’s hand (Figure 2) [5]. The percentages of the durations of the five phases were calculated as the ratio of each phase’s duration to total throwing time (i.e., from the instant of maximum backswing to the instant the discus releases from the participant’s hand). 

### 2.4. Statistical Analysis

SPSS 18 was used for data analysis. Using basic descriptive statistics (means and standard deviations) for all kinematic parameters and Pearson product-moment correlation coefficient (*r*) to understand the relationship between kinematic parameters and throwing distance. Coefficient of variation (*CV*) was calculated to look into variability of kinematic parameters. Low variability was defined as *CV* < 20; medium variability was defined as 20–40; and high variability was defined as *CV* > 40 [12]. The significance level was set at *p* < 0.05.

## 3. Results

In basic kinematics, the release velocity had high positive correlation with the throw distance (*r* = 0.952) (low *CV* values); the single-support phase (%) had high positive correlation with the throw distance (*r* = 0.782) (low *CV* values) (Table 1).

In the shoulder–hip separation angle, the instant of the right foot landing had high negative correlation with the throw distance (*r* = −0.810) (high *CV* values); the instant of the left foot landing had high negative correlation with the throw distance (*r* = −0.714) (high *CV* values) (Table 2).

In the throwing arm elevation angle, the instant of maximum backswing had high positive correlation with the throw distance (*r* = 0.857) (medium *CV* values); the instant of the right foot leaving the ground had high positive correlation with the throw distance (*r* = 0.810) (medium *CV* values); the instant of the left foot leaving the ground had high positive correlation with the throw distance (*r* = 0.833) (medium *CV* values); the instant of the right foot landing had high positive correlation with the throw distance (*r* = 0.833) (medium *CV* values) (Table 2).

## 4. Discussion

This is the first study to analyse the jumping discus throwing technique specifically in kinematics. The following findings were observed in the jumping discus throws with better performances. The body demonstrated a so-called X-type posture since the shoulder and hip twisting with the right ASIS was in front of the right acromion while the left ASIS was behind the left acromion until the instant of discus release for the right-handed thrower. The throwing movement of participants with better performances was practiced rhythmically in the total throwing time that was observed relatively short (fast movement) in the double-support phase, long (slow movement) in the single-support phase, short (fast movement) in the non-support phase, short (fast movement) in the second single-support phase, and short (fast movement) in the delivery phase. The discus was kept in a depressed position that was lower than the acromion.

Discus throwers’ performance is mainly determined by their throwing distance, which can be affected by release height, velocity, and angle [13,14,15,16], and the result of the current study showed that the release velocity had high positive correlation with the throw distance (*r* = 0.952) (low *CV* values). On average, world-leading male discus throwers can achieve a throwing distance of 64.17–66.76 m, release velocity of 23.88–25.71 m/s, release angle of 32.02°–37.23° [8,17,18,19,20], and release height of 1.4–2.0 m [9]. In this study, the participants achieved an average throwing distance of 42.08 m, average release velocity of 19.89 m/s, average release height of 1.69 m, and average release angle of 38.50°. A study identified 35°–44° as the optimal release angle [21], but throwing distance is primarily affected by angle of attack and release velocity [3,6]. Given that the participants achieved comparable release angle and height to those of world-leading athletes, the reason for their failure to achieve comparable throwing distance should mainly be attributed to their inferior release velocity in the current study.

The total throwing time was averaged 1.61 s in the current study, whereas that reported in previous studies was 1.40–1.42 s [9,17]. The time spent on each phase of discus throwing indicates an athlete’s movement coordination [6]. Compared with previous studies, the difference lies mainly in the double-support phase (0.69 s in this study and 0.57 s in other studies) and the initial single-support phase (0.4–0.42 s in this study and 0.37 s in other studies) [9,17], and the results of the current study showed that the double-support phase had no correlation with the throw distance, but the single-support phase (%) had high positive correlation with the throw distance.

When athletes transit from the double-support phase to the initial single-support phase, their right foot generates a pull force, while their left foot generates a push force, causing the body to swing to counter these forces [5]. Subsequently, the transition from the initial single-support phase to the non-support phase requires the thrower-plus-discus system because the trunk needs to generate rotary momentum of the system on the *Z* axis (vertical upward direction); insufficient rotary momentum of the system can cause the trunk, which is spinning in the air, to prematurely transit from the non-support phase to the second single-support phase, lengthening subsequent phases and compromising throwing distance [5,6,7,9]. The duration of the non-support phase (0.08 s) was close to that presented in previous studies, but it corresponded to a smaller percentage (5.13%) of total throwing time compared with previous studies that accounts for 6.0–6.64% of total throwing time [9,17], although the current study showed that the non-support phase had no correlation with the throw distance.

We found that the times in the double-support phase (present study: 0.69 s; previous studies: 0.57 s) and single-support phase (present study: 0.41 s; previous studies: 0.37 s) in the present study are longer than those in previous studies for total time (present study 1.61 s; previous studies: 1.40~1.42 s). However, the time spent in the non-support phase is shorter than other phases, but the second single support phase (present study: 0.20 s; previous studies: 0.18~0.19 s) and delivery phase (present study: 0.24 s; previous studies: 0.19~0.20 s) were longer than those in previous studies [9,17]. Therefore, the longer duration from the maximum backswing to the left toe off instant makes the thrower use rapid speed into the non-support phase, but it creates a long time in the second-single support phase and delivery phase as well as decreases the technique efficiently in the discus throwing [5,6,7,9].

In discus throw, the spinning of the body is the main source of energy for the discus. From the instant of maximum backswing to the instant of release, the body is in a constant twisting movement, with the right ASIS being in front of the right acromion (i.e., the shoulder–hip separation angle showed positive value). In the current study, the discus was nearly parallel to the right acromion at the instant the right foot landed; it showed the largest shoulder–hip twisting at the instant of the left foot landed.

The right acromion is in front of the right ASIS (i.e., the shoulder–hip separation angle showed negative value) at the instant of release. Previous studies have revealed that the upper limbs must generate smaller shoulder–hip twisting, with the right ASIS being in front of the right acromion in the initial single-support phase [9]. The shoulder–hip twisting observed in this study (15.68°) was greater in range than that reported in other studies (approximately −2°–14°) [9]. This is because the body, which is in a neutral position, spins extremely fast and requires smaller shoulder–hip twisting to maintain balance and rotational speed [22]. Moreover, smaller shoulder–hip twisting can help increase discus throwing distance [23].

In the delivery phase, the thrower must execute an explosive extension and internal rotation movement of the right hip and an extension movement of the left knee to enhance the left foot’s backward–upward force, thereby transmitting more energy from the lower limbs to the trunk [5,18]. At the same time, the energy generated through shoulder–hip twisting is transmitted through the throwing arm to the discus with the help of the angular velocity of the arm’s horizontal abduction to horizontal adduction movement, giving it the momentum to fly [22]. In terms of the arm–shoulder separation angle, the throwing arm was behind the shoulder from the instant of maximum backswing to the instant the left foot landed, and it only moved to the front at the instant of release. Regarding the throwing arm elevation angle, athletes kept the discus in a depressed position before the instant the left foot landed, and it cannot be higher than the acromion until the instant of release; meanwhile, the trunk tilted backward at the instant of release. At the instant of release, the movement of the throwing arm changed drastically from horizontal abduction to horizontal adduction, transferring the angular momentum of the thrower-plus-discus system generated by the swinging arm onto the discus to enable its flight [9,22].

There were limitations in the current study. The number of participants was small. Participants’ performance level was different from previous studies. Although technique is essential, strength is another key factor for enhancing release velocity [9]. However, subjects’ strength was not measured in the current study.

## 5. Conclusions

The current study demonstrated comprehensive kinematical analyses, which can be used to instruct the jumping discus throwing technique with duration and angle characteristics of throwing movement for throwers by athletes with videos (suitable for males). The shoulder–hip separation angle, arm–shoulder separation angle, and throwing arm elevation angle were key factors for jumping discus throw. Throwers should improve the efficiency of their technique by keeping the right ASIS in front of the right acromion (if the right-handed) from the instant the right foot lands to the instant the left foot lands (i.e., the single-support phase); keeping the discus in a depressed position, higher than the acromion; and increasing the time for the single-support phase, reducing the time before discus release and total throwing time in order to improve the performance. Additionally, release velocity must be improved because throwing distance is directly proportional to the squared release velocity.

## Figures and Tables

**Table 1:** Basic kinematics and duration parameters of the phases.

				*N* = 8
	*Mean* ± *SD*	*p*	*r*	*CV*
Basic kinematics				
Throw distance (m)	42.08 ± 4.89	-	-	low
Release height (m)	1.69 ± 0.15	0.823	0.095	low
Release velocity (m/s)	19.89 ± 1.13	0.000	0.952 **	low
Release angle (deg)	38.50 ± 3.35	0.779	0.119	low
Total throwing time (s)	1.61 ± 0.24	0.168	−0.539	low
Duration of phases				
Double-support phase (s)	0.69 ± 0.20	0.071	−0.667	medium
Single-support phase (s)	0.41 ± 0.03	0.910	−0.048	low
Non-support phase (s)	0.08 ± 0.02	0.154	−0.554	medium
Second single-support phase (s)	0.20 ± 0.02	0.629	0.204	low
Delivery phase (s)	0.24 ± 0.03	0.099	−0.623	low
Percentage of the duration of phases				
Double-support phase (%)	41.81 ± 8.14	0.937	0.027	low
Single-support phase (%)	25.79 ± 4.27	0.004	0.782 **	low
Non-support phase (%)	5.13 ± 0.85	0.709	−0.127	low
Second single-support phase (%)	12.75 ± 3.5	0.272	0.364	medium
Delivery phase (%)	14.99 ± 2.67	0.484	0.236	low

Note: ** *p* < 0.01; Low variability was defined as *CV* < 20; medium variability was defined as 20–40; and high variability was defined as *CV* > 40.

**Table 2:** Angle parameters at the instant of the throwing movements.

			*N* = 8	
	*Mean* ± *SD*	*p*	*r*	*CV*
Shoulder–hip separation angle (deg)				
Instant of maximum backswing	68.41 ± 13.25	0.320	0.405	low
Instant the right foot leaves the ground	35.39 ± 14.81	0.289	−0.429	high
Instant the left foot leaves the ground	19.71 ± 10.55	0.531	−0.262	high
Instant the right foot lands	21.60 ± 15.57	0.015	−0.810 *	high
Instant the left foot lands	44.04 ± 19.04	0.047	−0.714 *	high
Instant of release	−13.18 ± 6.62	0.570	0.238	high
Arm–shoulder separation angle (deg)				
Instant of maximum backswing	23.50 ± 17.62	0.955	−0.024	high
Instant the right foot leaves the ground	37.64 ± 7.16	0.651	−0.190	low
Instant the left foot leaves the ground	21.90 ± 8.57	0.736	0.143	high
Instant the right foot lands	32.11 ± 10.23	0.736	0.143	high
Instant the left foot lands	43.18 ± 7.48	0.693	−0.167	low
Instant of release	−2.46 ± 9.54	0.779	0.119	high
Throwing arm elevation angle (deg)				
Instant of maximum backswing	−21.02 ± 4.50	0.007	0.857 **	medium
Instant the right foot leaves the ground	−35.31 ± 8.32	0.015	0.810 *	medium
Instant the left foot leaves the ground	−25.69 ± 7.81	0.010	0.833 *	medium
Instant the right foot lands	−18.13 ± 7.12	0.010	0.833 *	high
Instant the left foot lands	−30.07 ± 13.61	0.160	−0.548	high
Instant of release	8.06 ± 6.44	0.736	−0.143	high
Trunk forward-backward tilt angle (deg)				
Instant of maximum backswing	16.66 ± 7.08	0.96	0.02	high
Instant the right foot leaves the ground	10.29 ± 9.47	0.96	0.02	high
Instant the left foot leaves the ground	10.45 ± 9.67	0.65	0.19	high
Instant the right foot lands	19.37 ± 6.94	0.32	0.40	medium
Instant the left foot lands	3.57 ± 10.13	0.35	0.38	high
Instant of release	−23.21 ± 5.65	0.18	0.52	medium

Note: Shoulder–hip separation angle (+Turn backward/−Turn forward); Arm–shoulder separation angle (+Backward swing/−Forward swing); Throwing arm elevation angle (+right shoulder raised/−right shoulder depressed); Trunk forward–backward tilt angle (+forward/−backward); * *p* < 0.05; ** *p* < 0.01; Low variability was defined as *CV* < 20; medium variability was defined as 20–40; and high variability was defined as *CV* > 40.
